# Dynamic Hydrogel‐Based Cytomimetic Models for Chemical Information Conduction

**DOI:** 10.1002/smsc.202500032

**Published:** 2025-07-27

**Authors:** Tian Liu, Fen Li, Junlong Song, Kai Zhang

**Affiliations:** ^1^ Sustainable Materials and Chemistry Department of Wood Technology and Wood‐based Composites University of Göttingen D‐37077 Göttingen Germany; ^2^ Jiangsu Co‐Innovation Center for Efficient Processing and Utilization of Forest Resources and Joint International Research Lab of Lignocellulosic Functional Materials Nanjing Forestry University Nanjing 210037 China; ^3^ Biotechnology Center (Biotechnikum) University of Göttingen D‐37077 Göttingen Germany

**Keywords:** cascade reactions, cavitary hydrogel, chemical information communication, cytomimetic models

## Abstract

Micro‐ and nanoscale cytomimetic models are widely studied in catalysis, adhesion, compartmentalization, communication systems, and membrane regeneration considering the proximity effect. However, there are few reports of proximity effect on the chemical information communication behavior of large‐sized cytomimetic models at ultra‐long distances. Herein, cytomimetic models are prepared by encapsulating enzymes glucose oxidase (Gox), catalase (Cat), and horseradish peroxidase (HRP) in millimeter‐sized spherical cavity hydrogels via a facile and efficient one‐step method. Considering the proximity effect, the biocatalytic efficiency of the cascade reactions between two separated cavity hydrogels as cytomimetic models with corresponding enzymes is less than that carried out inside one cytomimetic model by 30.6%. Using them as two individual closed systems, Closed System I with cascade reactions by Gox and Cat can stabilize 66.7% of dissolved oxygen within 160 min at room temperature. In comparison, 14.5% less of the dissolved oxygen remains in Closed System II with cascade reactions by Gox and HRP within 150 min due to the low ability of cascade reaction to circulate dissolved oxygen. This study highlights a promising new route to design millimeter scale dynamic hydrogel‐based cytomimetic models for various events, such as biosensing and controlled delivery.

## Graphical abstract

The enzymes of glucose oxidase, catalase, and horseradish peroxidase are encapsulated into cavitary hydrogels as cytomimetic models to investigate the cascade reaction efficiency inside two closed systems while considering the circulating performance of dissolved oxygen and thermal stimulation. The closed system I, with the ability to circulate dissolved oxygen, performs better than the closed system II.

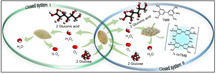

## 1 Introduction

Cells are the basic structural and functional unit of all known living organisms since Matthias Jakob Schleiden and Theodor Schwann proposed the cell theory in 1839.^[^ ^1^ ^]^ Cell biology research has been applied in many fields, such as the origin of life, cell engineering, biosensors, drug delivery, and medicines.^[^ ^2^ ^]^ To overcome the inherent complexity and the frangibility of natural cells, the concept of artificial cells was first proposed by Dr. Chang in 1957.^[^ ^3^ ^]^ Artificial cells are defined as a closed‐volume container designed and prepared using natural or synthetic materials that encapsulate bioactive substances like enzymes and gene sequences to keep bionic behavior.^[^ ^4^ ^]^ They exhibit functions similar to the biological processes of living cells, involving cell metabolism, cell communication, and biocatalytic degradation, which are used to study the characteristics and dynamics of natural cells with minimal interference from cellular complexity and explore new potential applications for alternative natural cells.^[^ ^5^ ^]^

Biological activities such as metabolism, signal transduction, cell proliferation, and immunoregulation occur based on a flux of energy and matter exchange,^[^ ^6^ ^]^ in which enzymatic cascade reactions play significant roles.^[^ ^7^ ^]^ In the cascade reactions, the product of an enzyme‐catalyzed reaction becomes the substrate for the following enzyme‐catalyzed reaction, forming a continuous reaction chain.^[^ ^7^ ^]^ Glucose oxidase (Gox), horseradish peroxidase (HRP), and catalase (Cat) are classic enzymes used for cascade reactions for in vitro mimicking bioinformation communication.^[^ ^8^ ^]^ Currently, research mainly focuses on the micro‐ and even nano‐level communication within artificial cells that are constructed with lipid/polymer, colloidosome, metal organic framework, and coacervates.^[^ ^5^, ^9^ ^]^ Due to its excellent biocompatibility and similar physical properties to cells, hydrogel is one of the most popular fundamental materials for constructing artificial cells.^[^ ^10^ ^]^ The natural extracellular matrix of an organization is a complex scaffold with different chemical and mechanical properties, which plays a crucial role in regulating important cellular functions. The soft and elastic hydrogels, maintaining the expansion state in water, can provide a three‐dimensional scaffold similar to the cell membrane in vivo through chemical design to support the growth and function of cells.^[^ ^11^ ^]^ This can be achieved through physical encapsulation, covalent conjugation, or electrostatic interactions.^[^ ^12^ ^]^ These intricate and complex models studied the chemical communication inside cells, which is restricted to the nanoscale and microscale.^[^ ^13^ ^]^ Many studies have reported that the closer the two enzymes used for cascade reaction were at the microscale or nanoscale, the higher the biocatalytic efficiency, known as the proximity effect.^[^ ^14^ ^]^

However, some chemical signals can be transported to a target by diffusion over large distances in an aqueous environment. For example, development, morphogenesis, tissue remodeling, and wound healing are typically driven by the orderly and directional migration of collective cells.^[^ ^15^ ^]^ This behavior is transmitted over long distances by the juxtacrine signal of cadherin. Apprehending such long‐range chemical communication is also significant for understanding life phenomena, the development of biomimetic networks, and new forms of chemical computing.^[^ ^16^ ^]^ Therefore, the transmission efficiency of chemical signals over long distances and how the proximity effect plays a role in long‐distance chemical communication or cascade reactions are important. However, there are scarce reports in this area.

Herein, the dynamic cavitary hydrogels fabricated with polyacrylamide or poly (N‐isopropyl acrylamide) were used as matrixes to encapsulate enzymes (Gox, HRP, and Cat) via an efficient one‐step method. Each cavitary hydrogel was loaded with one enzyme or two enzmyes as a cytomimetic model. Their stability and the spatiotemporal perception of the biocatalytic substrate in the external solution were investigated by detecting hydrogen peroxide and dissolved oxygen products. Furthermore, two closed systems were established to comparatively study the chemical information transmission and feedback behaviors between two cytomimetic models based on their abilities to perceive the intermediate product dissolved oxygen.

## 2 Results and Discussion

### 2.1 Fabrication and Characterization of Gel Enzymes

**Figure 1:**
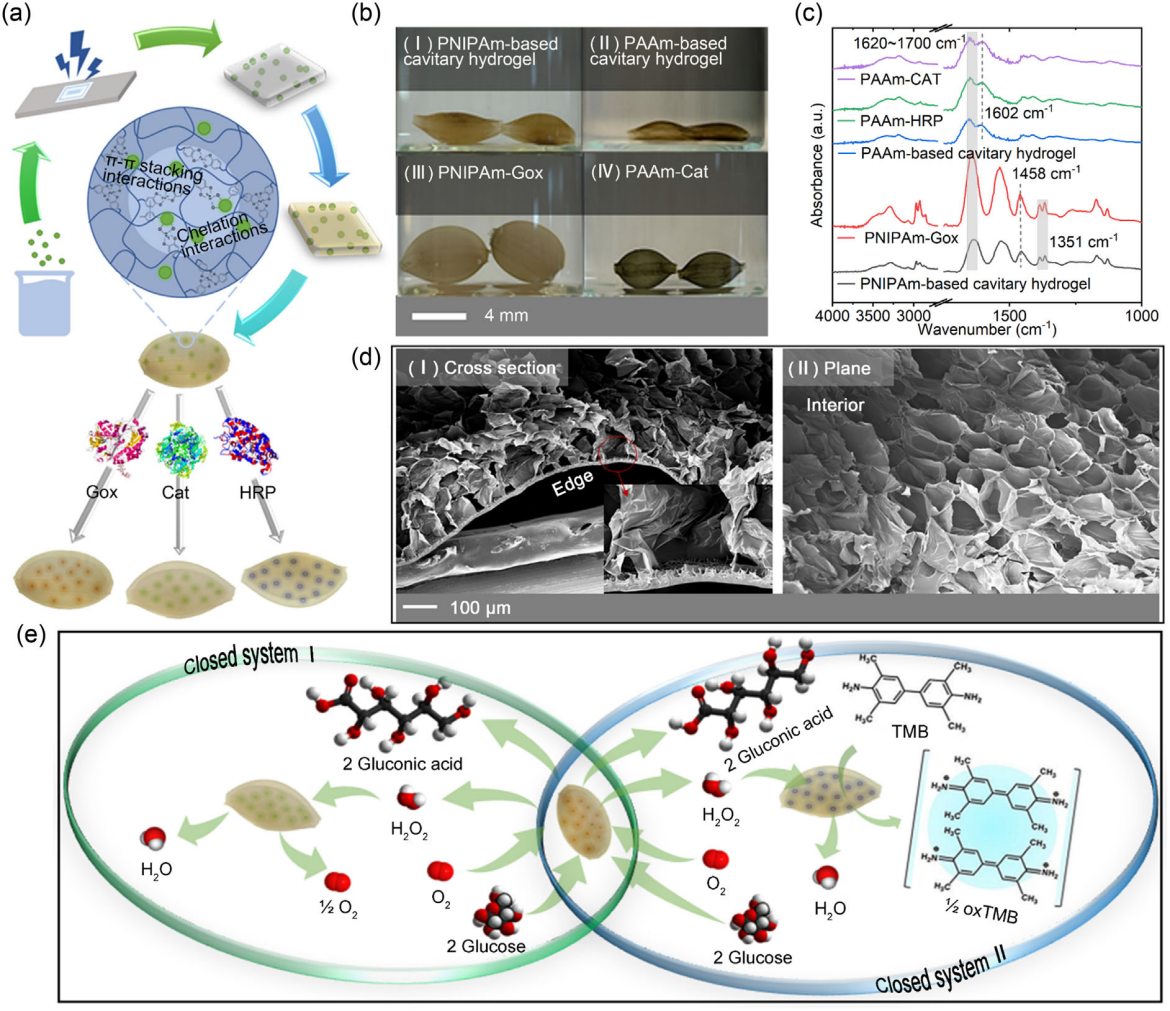
Fabrication and characterization of gels‐enzymes and two closed systems. a) Schematic diagram of a facile one‐step method for preparing enzyme‐loaded cavitary hydrogels; b) Macroscopic morphology of (I) PNIPAm‐based cavitary hydrogels, (II) PAAm‐based cavitary hydrogels, (III) PNIPAm‐Gox, (IV) PAAm‐Gox; c) FT‐IR spectra of cavitary hydrogels before and after loading enzymes; d) Microscopic morphology of (I) the cross‐section of PNIPAm‐based cavitary hydrogel and (II) the interior surface of PNIPAm‐based cavitary hydrogel; e) Two closed systems of the cascade reaction between PNIPAm‐Gox and PAAm‐Cat and the cascade reaction between PNIPAm‐Gox and PAAm‐HRP. The graph references protein structures from the PDB database: Gox (1CF3), Cat (1TGU), and HRP (1H58).

Cavitary hydrogels were designed and fabricated to encapsulate Gox, Cat, and HRP, respectively, as illustrated in **Figure** **1**a. The cavitary structure formed within 16 h due to the inner swelling based on the dissociation of dynamic‐covalent bonds in water and the protecting shell formed by metal‐coordinate bonds.^[11b]^ When the enzymes were encapsulated into the dynamic cavitary hydrogel, the hydrogel network offered steric hindrance to them. Additionally, π–π stacking interactions^[^ ^17^ ^]^ and chelation interactions between proteins and hydrogels favored encapsulation.

In this study, gels‐enzymes were obtained on a millimeter scale. Their sizes are easy to control, depending on the molds or equipment used for preparation. Under the same iron ion chelation time (30 s), the cavity structures formed by gel enzymes were more saturated than those of cavitary hydrogels without enzyme encapsulation. As shown in Figure 1b, the size of PNIPAm‐enzymes is 4 × 4 × 2.7 mm, while the size of PAAm‐enzymes is 6 ×6 × 4 mm. High inner osmotic pressure quickly drove the formation of the cavity structure of gel enzymes. Under the same preparation conditions, the cavity volume of PNIPAm‐enzymes was larger than that of PAAm‐enzymes. The FT‐IR spectra of the cavitary hydrogels before and after enzyme encapsulation were presented in Figure 1c. The vibration frequency of the amide I band ranged from 1600 ≈ 1700 cm^−1^ in dependence on the hydrogen bonding properties between C═O and N─H corresponding to the secondary structure formed between protein molecules and within molecules.^[^ ^18^ ^]^ The PAAm (PNIPAm)‐enzymes samples have stronger adsorption peaks within this wavelength range, implying the loading of enzymes. The peaks at 1351 and 1458 cm^−1^ belong to the vibrations of the isopropyl group, and those at 1602 and 3321 cm^−1^ due to the N─H stretching of the amide group in PNIPAm chains, which distinguish PAAm and PNIPAm.^[^ ^19^ ^]^ The triplet peaks at 2876, 2935, and 2972 cm^−1^ are attributed to C─H bands.^[^ ^20^ ^]^ The cross‐section and plane microscopic morphology of dried PNIPAm‐enzymes are shown in Figure 1d. The interior of the cavitary hydrogel is decorated with micrometer‐sized holes that provide ample space for adhering enzymes, while the exterior is like a dense shell that protects enzymes from harmful substances outside the cavity. At the same time, it would inhibit the substrates coming into the interior of gel enzymes and decrease their consumed amounts by enzymes.

Using these three types of gel enzymes, two closed systems were established to evaluate their communication efficiency via detecting the biocatalytic efficiency, as depicted in Figure 1e. In Closed System I, 2 mol of glucose consumed 1 mol of oxygen under the action of PNIPAm‐Gox to produce 2 mol of gluconic acid and 1 mol of hydrogen peroxide. Then, the 1 mol hydrogen peroxide was transformed by PAAm‐Cat into 1 mol of water and 0.5 mol of oxygen that would in turn be used by PNIPAm‐Gox. The oxygen circulated until the substrate was consumed entirely in Closed System I. In Closed System II, the difference was that the oxygen was not recycled. During the consumption of hydrogen peroxide by PAAm‐HRP, water molecules and oxygen anions were generated. The latter further oxidized 3,3′,5,5′‐tetramethylbenzidine (TMB), resulting in a blue color of the solution without generating oxygen.^[^ ^21^ ^]^

### 2.2 Distribution of Enzymes in Cavitary Hydrogels and Changes in Their Biocatalysis

**Figure 2:**
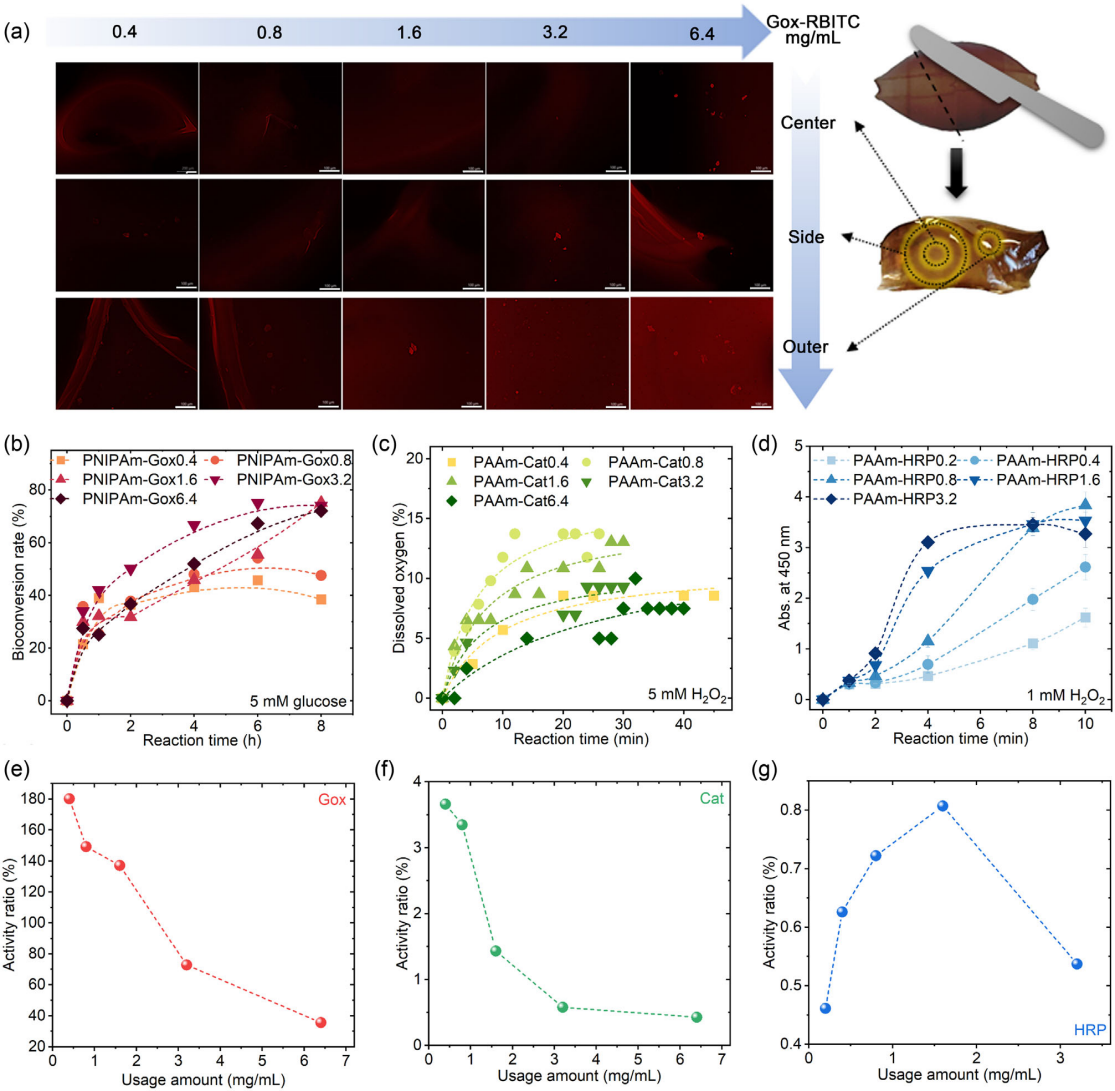
Distribution and relative bioactivity of enzymes in the cavitary hydrogels. a) Distribution of RBITC‐labeled Gox with different usage amounts in the cavitary hydrogel before use, the scale bar is 100 μm; b–d) Biocatalytic efficiency of PNIPAm‐Gox, PAAm‐Cat, and PAAm‐HRP with varying quantities of loading of enzymes; e–g) Calculated biocatalytic activity ratio of PNIPAm‐Gox to Gox with blue light illumination, PAAm‐Cat to Cat with blue light illumination and PAAm‐HRP to HRP with blue light illumination, respectively. The sample name gel ‐ enzmyes ‐ number in (b–d) represents the number mg/ml of enzyme loaded in the gel.

The distribution of enzymes in the cavitary hydrogel was investigated using PNIPAm‐Gox as a representative. The RBITC‐labelled PNIPAm‐Gox was cut into three parts: center, side, and outer to obtain the fluorescence images as shown in **Figure** **2**a. The average fluorescence intensity was shown in Table S1. With enzyme amount increased from 0.4 to 6.4 mg mL^−1^, the fluorescence intensity increased from 6.9 to 27.4 AU and 14.6 to 32.1 AU in the side part and outer part of PNIPAm‐Gox, respectively, while the fluorescence intensity reduced from 17.2 to 13.9 AU in the center part of PNIPAm‐Gox. So, the distribution of enzyme accumulated in the vicinity of the outer area as the enzyme loading increased from 0.4 to 6.4 mg mL^−1^. During the formation of the cavity structure, water flowed into the interior of the cavitary hydrogel. The high osmotic pressure inside the cavitary led to the tendency of the enzyme to move outward, which was blocked by the dense shell as the utmost layer of the hydrogels.

Substrate glucose was bio‐degraded by Gox to produce gluconic acid and hydrogen peroxide. The concentration of hydrogen peroxide is derived from the absorbance of a yellow‐colored complex formed by hydrogen peroxide with titanium oxysulfate, which can be determined by UV/VIS spectroscopy.^[^ ^22^ ^]^ The measured absorbance can be directly converted into the concentration of hydrogen peroxide according to a calibration equation using known hydrogen peroxide concentration as shown in Figure S1. Afterward, the intermediate hydrogen peroxide could be bio‐degraded by HRP into water and oxygen anion. At the same time, the latter could react with TMB to generate a blue‐colored complex determined via a similar photometric method.^[^ ^23^ ^]^ The reaction could be stopped by sulfuric acid, and the color of the solution changed from blue to yellow (Figure S2(a)). In comparison, the enzyme Cat bio‐degraded the hydrogen peroxide into water and oxygen, and no oxygen anion was generated to react with TMB. In this way, the generated dissolved oxygen was determined to evaluate the biocatalytic efficiency of PAAm‐Cat.

Compared to the biocatalytic efficiency of enzymes illuminated by blue light (Figure S3), the biocatalytic efficiency of PNIPAm‐Gox hardly decreased, while the biocatalytic efficiency almost reduced by an order of magnitude for PAAm‐Cat and PAAm‐HRP (Figure 2(b–d)). As shown in Figure S2(b) and Figure 2d, using PAAm or PNIPAm as a loading matrix had almost no effect on the biocatalytic efficiency. For the naked enzymes, the biocatalytic efficiency increased with higher enzyme dosage. However, when the dosage reached 6.4 mg mL^−1^, the biocatalytic efficiency slightly decreased because enzymes attached, reducing the mass transfer efficiency with substrates. For PNIPAm‐Gox and PAAm‐HRP, their biocatalytic efficiency reached the maximum after reacting for 8 h when the enzyme loading amount was more than or equal to 1.6 mg mL^−1^. In contrast, the PAAm‐Cat showed the best biocatalytic efficiency when the enzyme usage amount was 0.8 mg mL^−1^.

The PNIPAm‐Gox could not be degraded by strong acid or organic solvent, indicating that a solid interaction was generated between hydrogel and enzyme. By comparing the biocatalytic efficiency of the enzyme before and after loading into the cavitary hydrogel, relative enzyme activity is referred to as representing the biocatalytic efficiency of gel enzymes. The relative enzyme activities of gel enzymes are shown in Figure 2(e–g). All the biocatalytic reaction data were fitted by Equation (1) and Equation (2) to obtain the relationship between the bioconversion velocity and enzyme usage amount, and the details are presented in Figure S4–9. The relative enzyme activity of PNIPAm‐Gox and PAAm‐Cat descended from 180% to 35.6% and from 3.7% to 0.4%, respectively, with the usage amount increased.

In comparison, the relative enzyme activity of PAAm‐HRP augmented from 0.46% to 0.81%, with the usage amount increasing from 0.2 to 1.6 mg mL^−1^, and then it began to decrease. Among these three gel enzymes, PNIPAm‐Gox remained the maximum relative activity. It indicated that the enzyme loading amount generally had a downward trend with increased enzyme usage. On the other hand, the enzymes in the cavity had a spatiotemporal characteristic when they contacted the substrate solution penetrated from the outside due to the mass transfer resistance caused by the hydrogel matrix. All these factors led to a low bioconversion rate compared to the free enzymes after blue light illumination. Despite the PAAm‐Cat and PAAm‐HRP demonstrating deficient relative activity based on the free enzyme activity after blue light illumination, it did not affect their subsequent application, considering the increase in overall enzyme dosage for each gel enzyme.

### 2.3 Biocatalytic Efficiency of PNIPAm‐Gox

**Figure 3:**
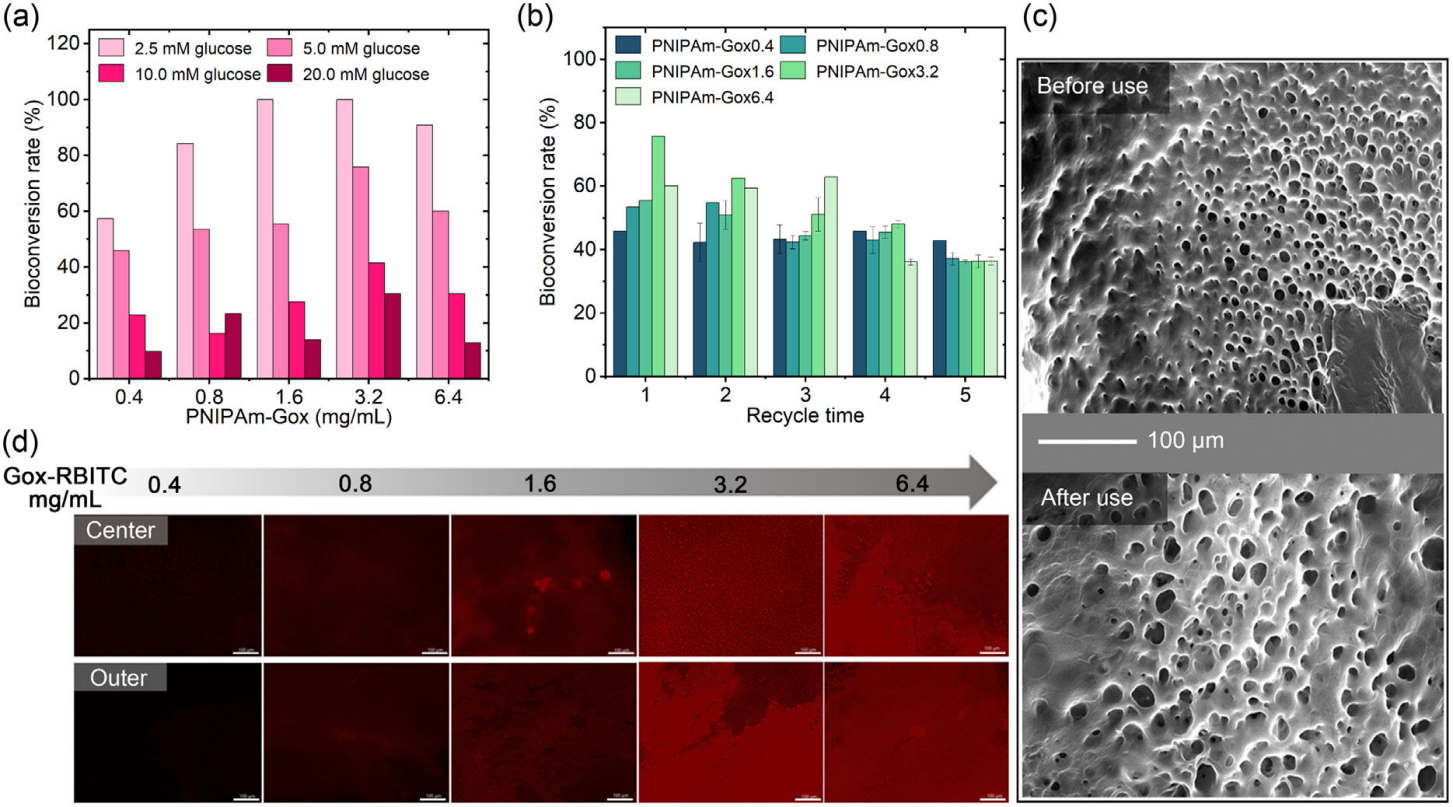
Biocatalytic efficiency of PNIPAm‐Gox. a) Biocatalytic efficiency of PNIPAm‐Gox for 6 h with different loading amounts of enzymes in a variety of substrate concentrations; b) The recycling potential of PNIPAm‐Gox under the condition of 5 mM glucose and 6 h; c) The microscale morphology of PNIPAm‐Gox1.6 before and after use for 30 h under the condition of 5 mM glucose; d) The distribution of RBITC‐labeled Gox with different loading amounts in PNIPAm‐Gox after use for 6 h. The scale bar in d) is 100 μm. The sample name gel ‐ enzmyes ‐ number in (a,b) represents the number mg/ml of enzyme loaded in the gel.

Because of the central role of PNIPAm‐Gox in cytomimetic models, its biocatalytic efficiency was studied as an example in more detail. The bioconversion rate of PNIPAm‐Gox in correlation with glucose concentration and enzyme usage amount is shown in **Figure** **3**a. Substrate concentration was one of the most significant factors for this biocatalytic reaction. With glucose concentration increasing, the bioconversion rate was reduced for PNIPAm‐Gox, and relative enzyme activity decreased. Though the substrate of higher concentrations outside the cavitary hydrogels produced greater osmotic pressure to promote the transport of the substrate molecules into the interior, there were not enough enzymes in PNIPAm‐Gox to completely capture the substrate, resulting in a low bioconversion rate for PNIPAm‐Gox with low relative enzyme activity. Under the same glucose concentration condition, the bioconversion rate of PNIPAm‐Gox showed a trend of first increasing and then slightly decreasing as the overall enzyme dosage increased. Considering the glucose concentration and overall enzyme dosage, the bioconversion rates of PNIPAm‐Gox1.6 and PNIPAm‐Gox3.2 could reach the maximum, approaching 100% biocatalysis of 2.5 mM glucose.

These cytomimetic hydrogels’ sustainable features, including environmental resistance or stable biocatalytic activity, are crucial for their long‐term use. The recycling performance of a single PNIPAm‐Gox in a 5 mM glucose solution is exhibited in Figure 3b. The bioconversion rate of PNIPAm‐Gox0.4 almost did not change after five cycles of biocatalytic reactions. The bioconversion rate of PNIPAm‐Gox0.8 showed virtually no change in the second cycle of the biocatalytic reaction but decreased by 11% in the third cycle. The bioconversion rate of PNIPAm‐Gox1.6 decreased by 4.6% in the second cycle of biocatalytic response. It was further reduced by 6.5% in the third cycle, while the bioconversion rate of PNIPAm‐Gox3.2 decreased by ≈10% in both the second and third cycles of the biocatalytic reaction. For PNIPAm‐Gox6.4, the bioconversion rate remained almost unchanged in the third cycle of the biocatalytic reaction but decreased by 23.9% in the fourth cycle. Apart from PNIPAm‐Gox0.4, the bioconversion rate of PNIPAm‐Gox0.8 (1.6, 3.2, and 6.4) remained at 36% ≈37% at the fifth cycle of the biocatalytic reaction. It implied that PNIPAm‐Gox with a low enzyme amount showed better biocatalytic efficiency than the PNIPAm‐Gox with a high enzyme amount after five cycles of biocatalytic reactions. As for the PNIPAm‐Gox with higher enzyme usage, it lost more catalytic ability after being reused five times.

Moreover, the microstructure of PNIPAm‐Gox remained after five cycles, compared with the initial microstructure (Figure 3c), while only the pores were slightly enlarged. To visualize the Gox after 6 h reaction, the fluorescence images of PNIPAm‐Gox are shown in Figure 3d and the average fluorescence intensity was shown in Table S1. The fluorescence strengthened with the increase in enzyme amount. RBITC‐labeled Gox was evenly distributed in the cavity hydrogel, comparing the fluorescence in the center and outer parts of PNIPAm‐Gox after use. The enzyme distribution of PNIPAm‐Gox after use was very different from that of PNIPAm‐Gox before use. It was caused by turbulence of substrate penetrating the cavity and product exiting it.

### 2.4 The Difference Between the Enzymes Catalyzed Cascade Reaction Inside One Cavity Hydrogel and the Cascade Reaction Between Gel Enzymes

**Figure 4:**
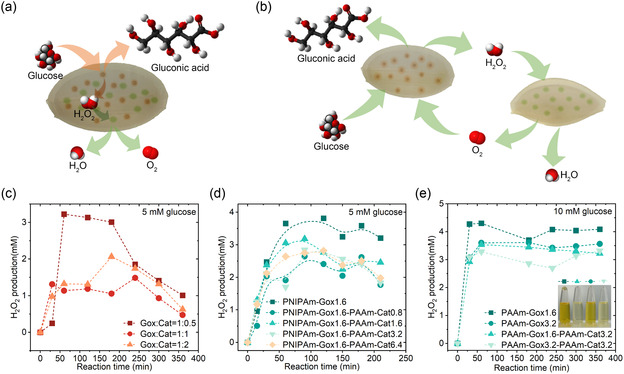
The differences of the cascade reactions inside cavitary hydrogel and between cavitary hydrogels. Schematic diagram of the substance flow routes between gel‐Gox and gel‐Cat a,b) and inside a cavitary hydrogel; c) The intermediate hydrogen peroxide production of a cascade reaction inside PNIPAm‐based cavitary hydrogel with different loading amount ratio of Gox to Cat at a concentration of 5 mM glucose; d) The intermediate hydrogen peroxide production of a cascade reaction between PNIPAm‐Gox1.6 and PAAm‐Cat0.8 (1.6, 3.2, and 6.4) at a concentration of 5 mM glucose; e) The intermediate hydrogen peroxide production of a cascade reaction between PAAm‐Gox1.6 (3.2) and PAAm‐Cat3.2 at a concentration of 10 mM glucose. All the experiments were conducted at room temperature. The sample name gel ‐ enzmyes ‐ number in (c–e) represents the number mg/ml of enzyme loaded in the gel.

A cascade reaction of Gox‐Cat was conducted in the PNIPAm‐based cavitary hydrogel, as depicted in **Figure** **4**a. In the interior part of the cavity structure, once the hydrogen peroxide was generated from glucose by Gox, it would be immediately consumed by nearby Cat. Then, the dissolved oxygen and water were produced and diffused outside the cavity structure. Excessively saturated hydrogen peroxide solution would penetrate the cavity structure and be detected. As shown in Figure 4c, the concentration of produced hydrogen peroxide obviously decreased from 3.22 to 1.14 mM at cascade reaction for 60 min when the loading amount ratio of Cat to Gox in one PNIPAm‐based cavitary hydrogel increased from 0.5 to 1.0. However, the concentration of hydrogen peroxide did not continuously decrease, even increased by about 0.2 mM at the same cascade reaction time when the loading ratio of Cat to Gox in the PNIPAm‐based cavitary hydrogel rose from 1 to 2.

A cascade reaction between gel‐Gox and gel‐Cat is shown in Figure 4b. The hydrogen peroxide needed to diffuse with the liquid and pass through the hydrogel wall of gel‐Cat again before it could be consumed by Cat inside, compared with the hydrogen peroxide consumption in Figure 4a. In general, the concentration of produced hydrogen peroxide decreased by a maximum of 1.75 mM when PNIPAm‐Gox1.6 and PAAm‐Cat0.8 underwent a cascade reaction. Compared with the cascade reaction inside the cavity hydrogel, the cascade reaction rate between the cavity hydrogels decreased by 30.6%. As shown in Figure 4e, hydrogen peroxide production reached 3.6 mM at the cascade reaction for 60 min when the glucose concentration rose to 10 mM for PAAm‐Gox1.6. At the same concentration of glucose, the hydrogen peroxide production was reduced to 3.6 mM for PAAm‐Gox3.2. The hydrogen peroxide production dropped by 0.76 mM for the cascade reaction between PAAm‐Gox1.6 and PAAm‐Cat3.2, while it dropped by 0.31 mM for the cascade reaction between PAAm‐Gox3.2 and PAAm‐Cat3.2. In brief, when the glucose concentration and enzyme loading amount increased, the intermediate product hydrogen peroxide consumption rate decreased.

### 2.5 Use of Gel Enzymes in Two Closed Systems

Bare enzymes of cascade reactions can be proceeded quickly and face various challenges, such as enzyme deactivation, difficult separation, and recycling of multiple enzymes. Enzyme compartmentalization using artificial cells can solve this problem.^[^ ^24^ ^]^ Simultaneously, when compartmentalized multiple enzymes are used for a cascade reaction, the proximity effect is significant due to diffusion limitation caused by compartments.^[^ ^13^, ^25^ ^]^ Most reports amplified cascade reactions and studied them at the micro and nanoscale to protect the original rate of cascade reactions.^[^ ^26^ ^]^ In contrast, a long‐range (> microscale) cascade reaction in sealed vessels was built in this study. Closed System I simulated a situation where the signal (dissolved oxygen) was produced and consumed between a cascade reaction without an extra oxygen supplement, and Closed System II imitated a situation where the signal was constantly consumed in a cascade reaction without an extra oxygen supplement.

**Figure 5:**
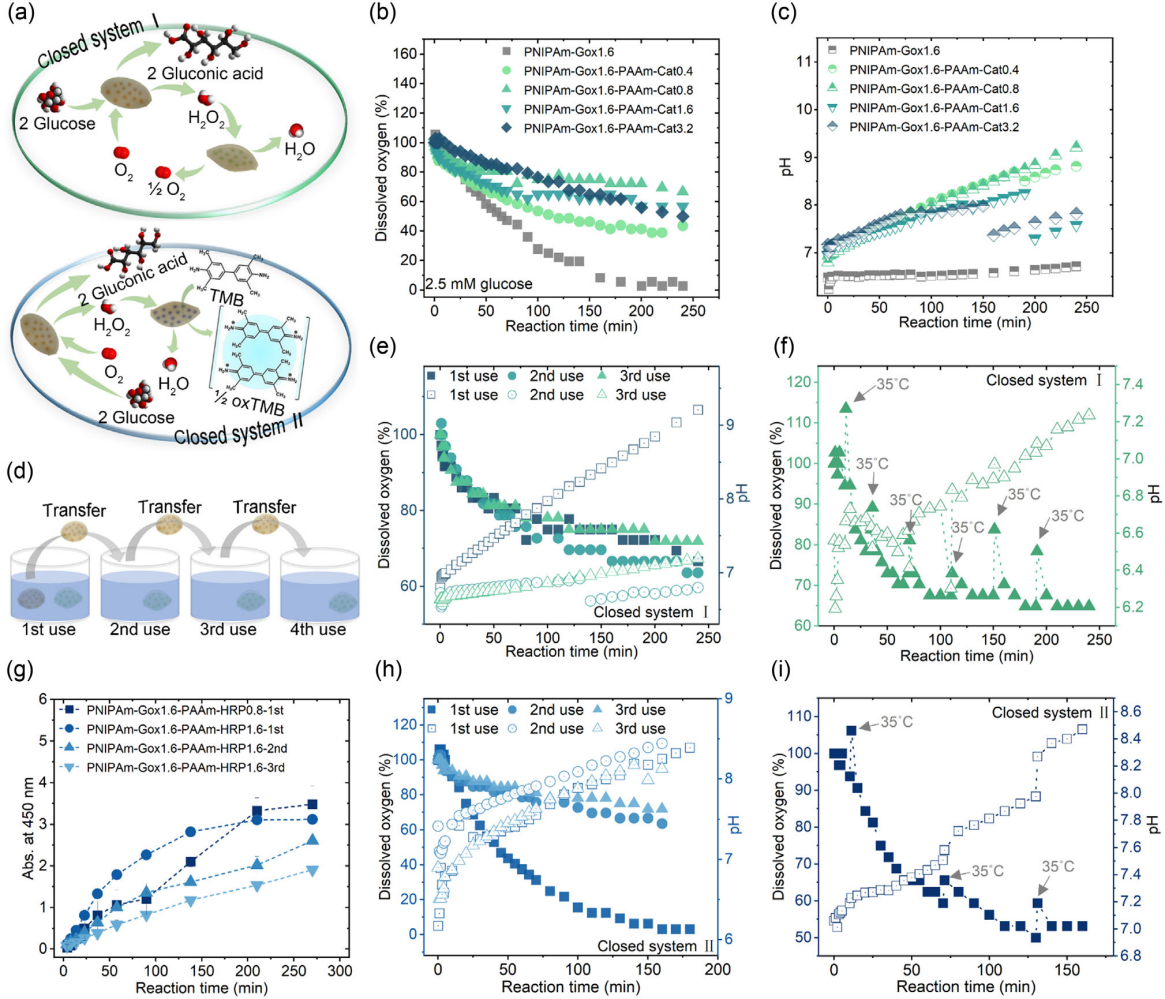
Use of cytomimetic hydrogels in two closed systems. a) Schematic diagram of the substance flow routes in these two closed systems; The change of b) the dissolved oxygen and c) pH during the cascade reaction between PNIPAm‐Gox1.6 and PAAm‐Cat in Closed System I; d) Schematic diagram of the recycling use for these two closed systems; e) Dissolved oxygen and pH in Closed System I after different cycles of PNIPAm‐Gox1.6; f) Thermal responsiveness of the cascade reaction between PNIPAm‐Gox1.6 and PAAm‐Cat0.8 in Closed System I; g) Biocatalytic efficiency of the cascade reaction between PNIPAm‐Gox1.6 and PAAm‐HRP in Closed System II; h) Dissolved oxygen and pH in Closed System II after different cycles of PNIPAm‐Gox1.6; i) Thermal responsiveness of the cascade reaction between PNIPAm‐Gox1.6 and PAAm‐HRP0.8 in Closed System II. (e–i) solid symbol represents dissolved oxygen and hollow symbol represents pH; The sample name gel ‐ enzmyes ‐ number represents the number mg/ml of enzyme loaded in the gel.

One PNIPAm‐Gox1.6 sample and one PAAm‐Cat sample were placed in a sealed vessel with 2.5 mM glucose solution, as in Closed System I (**Figure** **5**a), the consumption of dissolved oxygen within 250 min was recorded as shown in Figure 5b. The change of dissolved oxygen in a sealed vessel containing only one NIPAm‐Gox1.6 sample was used as a blank control. The dissolved oxygen was depleted within 200 min without an extra dissolved oxygen supplement. The PAAm‐Cat participated in the cascade reaction with PNIPAm‐Gox1.6 and prolonged reaction time, consuming intermediate hydrogen peroxide to produce dissolved oxygen, compensating for the consumption of dissolved oxygen for PNIPAm‐Gox1.6. Simultaneously, the consumption of hydrogen peroxide avoided its excessive accumulation and damaging enzyme activity. Within 250 min, the dissolved oxygen decreased by 33.3%, 43.2%, 50%, and 61%, respectively, for the cascade reaction of PNIPAm‐Gox1.6‐PAAm‐Cat0.8, PNIPAm‐Gox1.6‐PAAm‐Cat1.6, PNIPAm‐Gox1.6‐PAAm‐Cat3.2, and PNIPAm‐Gox1.6‐PAAm‐Cat0.4, respectively. The efficiency of dissolved oxygen supplied by PAAm‐Cat was as follows: PAAm‐Cat0.8 > PAAm‐Cat1.6 > PAAm‐Cat3.2 >PAAm‐Cat0.4. As shown in Figure 5c, with the cascade reaction proceeding in Closed System I, the pH increased due to hydrogen peroxide decomposition. The pH of the closed system containing only PNIPAm‐Gox1.6 hardly changed for the accumulation of hydrogen peroxide. The pH of PNIPAm‐Gox1.6‐PAAm‐Cat0.8 rose from 6.8 to 9.2, exhibiting the maximum change due to the rapid degradation of a large amount of hydrogen peroxide by PAAm‐Cat.

In Closed System II (Figure 5a), one PNIPAm‐Gox1.6 and one PAAm‐HRP, along with chromogenic agent TMB, were placed in a sealed vessel containing 2.5 mM glucose solution. The efficiency of the cascade reaction between PNIPAm‐Gox1.6 and PAAm‐HRP within 270 min in Closed System II is shown in Figure 5g. The cascade reaction efficiency of PNIPAm‐Gox1.6‐PAAm‐HRP0.8 reached the maximum absorbance value of 3.5 at 270 min, while the maximum absorbance value of 3.1 for PNIPAm‐Gox1.6‐PAAm‐HRP1.6 at 210 min. The maximum absorbance values of these two cascade reactions were equivalent to those reported for direct hydrogen peroxide degradation in Figure 2c. However, compared to the time required for the catalytic reaction of PAAm‐Cat to reach its maximum value, the time required for the cascade reaction in Closed System II to reach its maximum value increased by 25–26 times. The degradation of glucose by PNIPAm‐Gox1.6 generates hydrogen peroxide, and the diffusion of hydrogen peroxide from PNIPAm‐Gox1.6 to PAAm‐HRP requires time. Compared with the cascade reaction efficiency in nanoscale with a long‐range sensing‐feedback mechanism,^[^ ^27^ ^]^ the proximity effect seemed negligible. After using PNIPAm‐Gox1.6 once, it was recycled and placed again in a sealed vessel with a fresh PAAm‐HRP1.6 for cascade reaction, as depicted in Figure 5d. The cascade reaction efficiency decreased by 16% and 27% for the second and third cascade reactions, respectively, compared to the reaction efficiency of the first cascade reaction.

All the recycling experiments were conducted, as shown in Figure 5d. Each sealed vessel contained 2.5 mM glucose solution and a fresh PAAm‐Cat or PAAm‐HRP. After the previous cascade reaction was completed, the PNIPAm‐Gox1.6 was rinsed with deionized water and placed in the next sealed reaction vessel. The variation of dissolved oxygen and pH both in Closed System I and Closed System II are shown in Figures 5e and (h), respectively. For Closed System I, the dissolved oxygen and pH detected in the second and third cascade reactions were almost maintained compared to the first, while the pH had less increment. In the first use, the pH increased from 6.5 to 9.2 due to the quick decomposition of hydrogen peroxide. Still, the increase in pH was less than 1.0 in the second and third use, implying that the biocatalytic efficiency of PNIPAm‐Gox1.6 decreased and less intermediate hydrogen peroxide was generated to provide for PAAm‐Cat0.8. PNIPAm‐Gox1.6 consumed less dissolved oxygen and less dissolved oxygen was generated by PAAm‐Cat0.8. For Closed System II, the dissolved oxygen detected in the second use decreased by 60.5% compared to the first use, implying the biocatalytic efficiency of Closed System II was greatly reduced. The consumption rate of dissolved oxygen slowed down. And the dissolved oxygen tested in the third‐time use decreased by 8.3% based on the second‐time use. Correspondingly, the pH rose from 7.5 to 8.4, 7.9 to 8.4, and 7.6 to 8.1 in the first, second, and third cascade reactions, respectively. It suggested that the biocatalytic reaction in Closed System II was sensitive and mild. Comparing these two closed systems, the dissolved oxygen was easy to balance, and the pH was sensitive to the environment in Closed System I, as well as the dissolved oxygen was sensitive to the environment, and the pH was easy to balance in Closed System II.

Thermo‐responsiveness of Closed System I and Closed System II are exhibited in Figures 5f and (i), respectively. The cascade reaction proceeded for 210 min in Closed System I while the cascade reaction proceeded for 140 min in Closed System II. When the reaction temperature improved to 35 °C, the PNIPAm‐Gox1.6 shrunk to discharge the liquid containing hydrogen peroxide and gluconic acid from the cavitary hydrogel. Then, the hydrogen peroxide was rapidly biodegraded by PAAm‐Cat to generate dissolved oxygen and water. So, the detected dissolved oxygen increased, and the pH decreased. When the reaction temperature recovered to room temperature, the PNIPAm‐Gox1.6 expanded and inhaled the substrate solution to promote the reaction inside the cavitary hydrogel. Even though Closed System I repeatedly heated up and cooled down twice as many times as Closed System II, the dissolved oxygen was reduced by 35.1% in Closed System I, comparable to the value without heating. The dissolved oxygen was reduced by 46.9% in Closed System II compared to the depleted dissolved oxygen for the first‐time use shown in Figure 5h. It was caused by the shrinking cavity hydrogel network, which prevented the substrate solution from entering the cavity to participate in the cascade reaction, thus reducing the biocatalytic efficiency of the closed system. However, the pH rose from 6.2 to 7.2 and 7.0 to 8.5 for Closed System I and Closed System II, respectively, indicating the decomposition of more intermediate in Closed System II than in Closed System I.

## 3 Conclusion

This study used dynamic cavitary hydrogels with encapsulated Gox, Cat, and HRP as cytomimetic models, which were fabricated via a one‐step method. The millimeter‐scale individual cavity hydrogels mimicking artificial cells exhibited comparable biocatalytic efficiency under blue light exposure at different enzyme loadings. Through fluorescence tracking, the Gox in PNIPAm‐Gox was distributed more evenly in the cavity after use. The biocatalytic efficiency of PNIPAm‐Gox with a lower enzyme loading amount remained almost unchanged after five reaction cycles. Compared with the efficiency of the cascade reaction between Gox and Cat in one PNIPAm‐based cavitary hydrogel, the efficiency of the cascade reaction between PNIPAm‐Gox and PAAm‐Cat reduced by 30.6%. Then, the cascade reactions PNIPAm‐Gox‐PAAm‐Cat and PNIPAm‐Gox‐PAAm‐HRP‐TMB were established for two closed systems to evaluate the chemical communication between individual artificial cells. Compared to Closed System II, the chemical communication ability of Closed System I was good after three cycles of cascade reaction. The dissolved oxygen was consumed entirely in Closed System II, while the dissolved oxygen consumption reached 25% in Closed System I within 160 min. These two closed systems could control the substrate and product to enter and exit the cavitary hydrogel based on the response to temperature (35 °C). Closed System I responded once more than Closed System II within 150 min, while the remaining dissolved oxygen in Closed System I was higher than Closed System II by 14.5%. Even at extremely long distances (>microscale), individual artificial cells had remarkable chemical signal sensing capabilities between them, implying the proximity effect can be ignored. It paves a novel way for designing millimeter‐scale cytomimetic models in biosensing applications. Moreover, further investigation of the impact of reducing gel enzyme size on biocatalytic efficiency and whether gel enzyme is suitable for cascade reactions of other types of enzymes are significant.

## 4 Experimental Section

4.1

4.1.1

##### Materials

Acrylamide (AAm), N‐isopropyl acrylamide (NIPAm), 3‐(acrylamido) phenylboronic acid (PBAAm), dopamine methylacrylate (DMA), iron (III) chloride hexahydrate, glucose, glucose oxidase from Aspergillus niger, catalase, horseradish peroxidase, TMB, and titanium oxide sulfate were purchased from Sigma‐Aldrich. Lithium phenyl‐2,4,6‐trimethylbenzoylphosphinate (LAP) was provided by Yuanye Corp.

##### One‐Step Fabrication of Cavity Hydrogels Loaded with Enzymes

The method to prepare dynamic hydrogels was according to Zhang et al.^[11b]^ Solution of AAm (2 M) or NIPAm (2 M), PBAAm (0.025 M), and dopamine methylacrylate (0.025 M) were mixed with borax‐NaOH buffer (pH 10) to form the gel precursor. Enzyme solutions with different concentrations of phosphate buffer were mixed with hydrogel precursor. Subsequently, the mixed solution added with 0.5 wt% of initiator LAP was transferred into a mold with a size 4 × 4 × 1 mm, and exposed to blue light (380 ≈ 420 nm) with an intensity of 8 W cm^−2^ for 30 min to finish the photopolymerization and gelation process. Then, the dynamic hydrogels embedded with enzymes were immersed in 0.1 M of ferric chloride aqueous solution for 30 s. Afterward, the samples were placed into deionized water to form a cavitary structure and remove impurities. The specimens made of PAAm or PNIPAm and loaded with different enzymes (Gox, Cat, and HRP) were named gel (PAAm or PNIPAm)‐enzyme (Gox, Cat, or HRP).

##### Evaluation of the Biocatalytic Efficiency of Gel Enzymes

Glucose solution (5 mM) was used as the substrate for PAAm‐Gox encapsulated with different concentrations of Gox. As the reaction proceeded, 0.1 mL of sample solution was mixed with 0.2 mL of titanium oxide sulfate at regular intervals to test the absorbance change at 407 nm via a photometric method. The biocatalytic ability of PAAm‐Cat was carried out in 5 mM hydrogen peroxide to test the consumption of dissolved oxygen by a multi‐functional dissolved oxygen meter (WTW, Germany). The biocatalytic ability of PAAm‐HRP was carried out in 1 mM of hydrogen peroxide with adding TMB as a chromogenic agent. At regular intervals, a 1 mL sample solution was mixed with 0.5 M H_2_SO_4_ to stop the reaction, and the absorbance was tested at 450 nm via the photometric method. All the biocatalytic experiments were conducted in sealed vessels at room temperature. Then, the biocatalytic ability of PAAm‐enzymes based on the comparison of naked enzyme activity, also named relative enzyme activity, was fitted by the Michaelis–Menten model Equation (1) and Dose‐Response model Equation (2) as shown below

(1) $$y=\frac{v_{max}\cdot x}{k_{m}+x}$$

where the *v*_*max*_ was defined as the maximum production, *k*_*m*_ was defined as the reaction time for producing 1/2 *v*_*max*_.

(2) $$y=A1+\frac{A2-A1}{1+10^{(logx_{0}-x)\cdot p}}$$

where 1/2 (A1+A2) was defined as the product volume at maximum production speed, and *logx*_*0*_ was defined as the time it takes to produce the product at the maximum speed.

The enzymes (Gox, Cat, and HRP) with the same loading amount were not loaded onto the cavitary hydrogel but were directly exposed under blue light for the same photopolymerization time. Then, the biocatalytic efficiency of the enzymes after blue light exposure was detected using the same method and conditions as the blank control for calculating the biocatalytic activity of gel enzymes.

##### Cascade Reaction Efficiency in Two Closed Systems

Closed System I contained a cascade reaction between PNIPAm‐Gox and PAAm‐Cat. The change of intermediate products, including dissolved oxygen and gluconic acid (reflection as a change in pH) was monitored by a multi‐functional dissolved oxygen meter (WTW, Germany). Closed System II had a cascade reaction between PNIPAm‐Gox and PAAm‐HRP. The chromogenic agent TMB was used to reflect the change of hydrogen peroxide in this system. In addition, the recycling potential of these two closed systems was assessed by measuring the variety of dissolved oxygen and pH generated by repeated use of PNIPAm‐Gox. The thermal responsiveness of these two closed systems was evaluated by heating them at 35 °C for 20 s until a significant phase transition occurred in PNIPAm‐Gox, then cooling them at room temperature for different durations until the PNIPAm‐Gox returned to its original state.

##### Characterizations

The micro‐morphology of PNIPAm‐based cavitary hydrogel was observed by scanning electron microscopy (SEM) with a LEO Supra‐35 high‐resolution field emission scanning electron microscope (Carl Zeiss AG, Germany) or an EVO LS15 scanning electron microscope (Carl Zeiss AG, Germany) at an accelerating voltage of 5 kV. FT‐IR spectroscopy (Alpha, Bruker, USA) analyzed the composition changes of PAAm (PNIPAm)‐based cavitary hydrogels before and after encapsulating enzyme by pellet method. The UV/VIS spectrometer (Lambda 25, PerkinElmer, USA) was used to test the absorbance. The distribution of enzymes with RBITC‐label in the cavitary hydrogel was observed by a confocal fluorescence microscope (Keyence, Germany). Gox, as a representative enzyme, first reacted with Rhodamine B isothiocyanate (RBITC) in the dark at room temperature for 3 h and then at 4 °C for 12 h.^[^ ^28^ ^]^ Subsequently, the mixed solution was dialyzed (MWCO 1000) in deionized water to remove the free RBITC molecules. The dried RBITC‐labeled Gox was obtained by freeze‐drying.

## Conflict of Interest

The authors declare no conflict of interest.

## Supporting information

Supplementary Material

## Data Availability

The data that support the findings of this study are available from the corresponding author upon reasonable request.
