# Supplementary material for: Dynamic Hydrogel‐Based Cytomimetic Models for Chemical Information Conduction
Source: Small Sci. 2025 Jul 27;5(10):2500032. doi: 10.1002/smsc.202500032 (PMC12499378; doi:10.1002/smsc.202500032)
Supplement: Supplementary file 1 — Supplementary Material [file SMSC-5-2500032-s001.pdf]

Dynamic Hydrogel-based Cytomimetic Models for Chemical Information Conduction

Tian Liu, Fen Li, Junlong Song, and Kai Zhang**

T. Liu, F. Li, K. Zhang

Sustainable Materials and Chemistry, Department of Wood Technology and Wood-based Composites, University of Göttingen, D-37077 Göttingen, Germany;

Email: kai.zhang@uni-goettingen.de

T. Liu, J. Song

Jiangsu Co-Innovation Center for Efficient Processing and Utilization of Forest Resources and Joint International Research Lab of Lignocellulosic Functional Materials, Nanjing Forestry University, Nanjing, 210037, China;

Email: junlong.song@njfu.edu.cn

K. Zhang

Biotechnology Center (Biotechnikum), University of Göttingen, D-37077 Göttingen, Germany;

Email: kai.zhang@uni-goettingen.de

Table S1. The average fluorescence intensity of different parts of PNIPAm-Gox-RBITC before and after use.

AU	Gox-RBITC 0.4 mg/mL	Gox-RBITC 0.8 mg/mL	Gox-RBITC 1.6 mg/mL	Gox-RBITC 3.2 mg/mL	Gox-RBITC 6.4 mg/mL
Before use-center	17.2	16.1	13.5	9.9	13.9
Before use-side	6.9	11.3	11.1	11.8	27.4
Before use-outer	14.6	14.1	19.9	23.8	32.1
After use-center	9.3	16.1	21.7	39.5	41.1
Afte use-outer	4.2	13.4	21.0	41.5	36.9

(a)

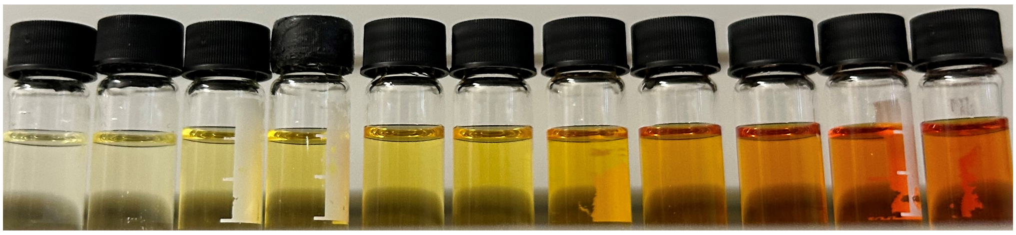

Color change from 0.001 M to 0.1 M (0.1 mL sample+ 0.2 mL $TiOSO_4$)

(b)

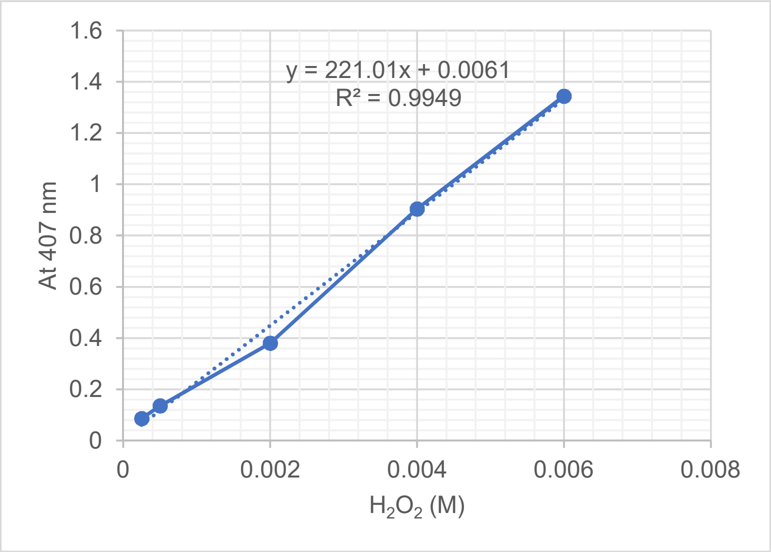

Figure S1. (a) Color change for 0.1 mL hydrogen peroxide with concentrations from 0.001 M to 0.1 M reacted with 0.2 mL titanium oxysulfate; (b) Hydrogen peroxide standard curve obtained by UV/VIS spectrometer.

(a)

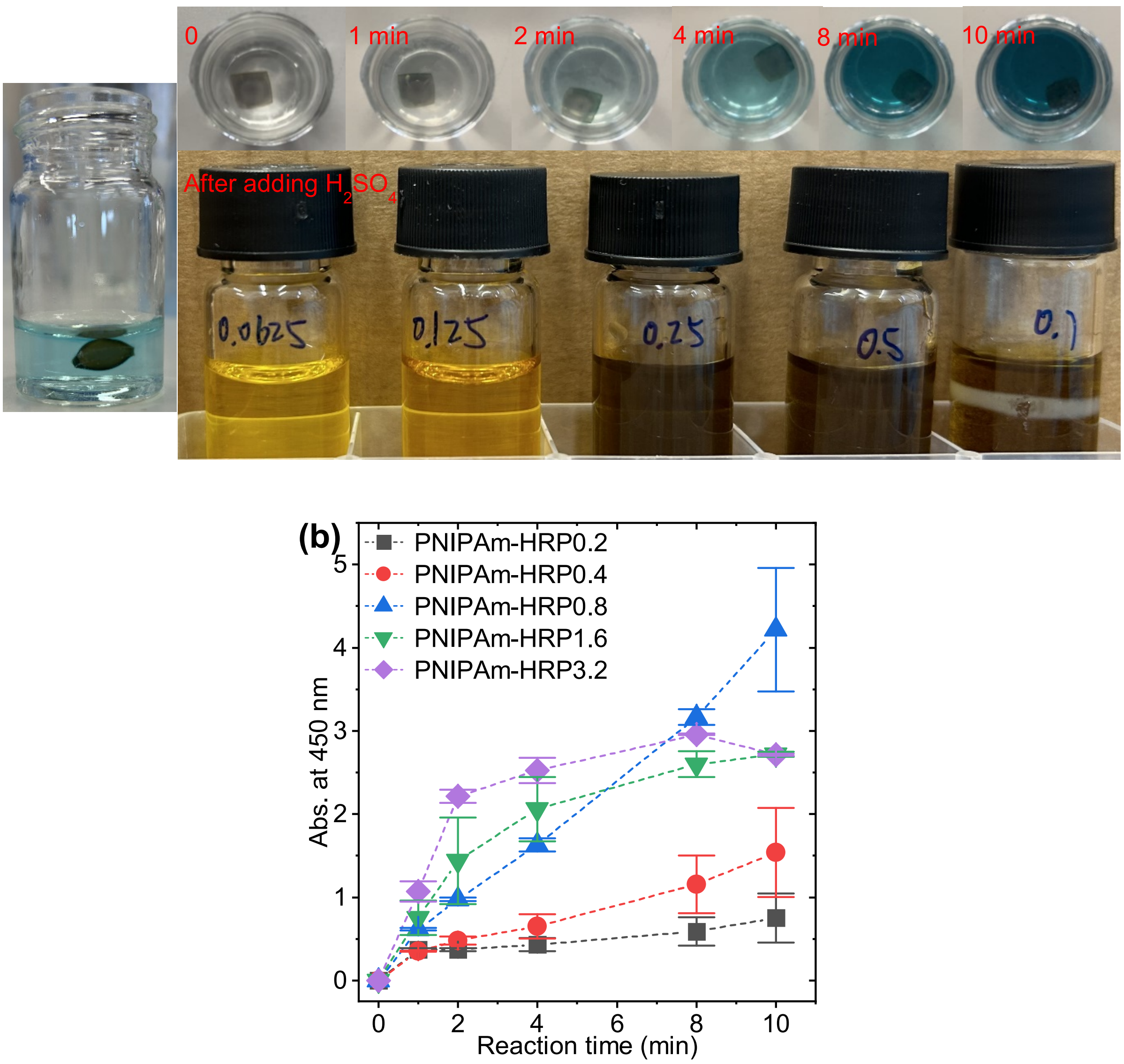

Figure S2. (a) Pictures of the biocatalysis process of PNIPAm-HRP; (b) The bioconversion rate of PNIPAm-HRP with different usage amount.

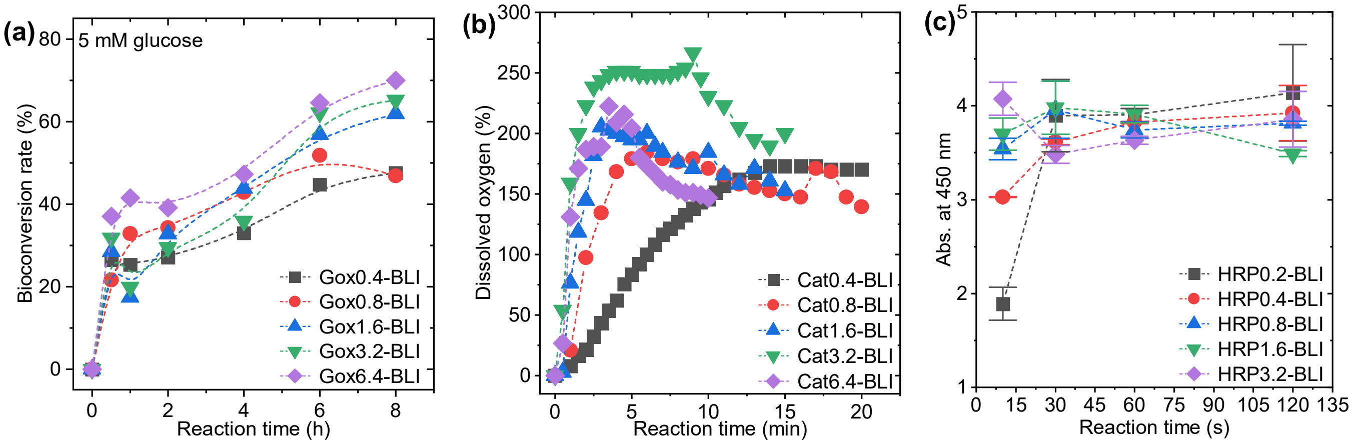

Figure S3. Bioconversion rate of (a) Gox-BLI, (b) Cat-BLI and (c) HRP-BLI with different usage amount. BLI represents blue light illumination.

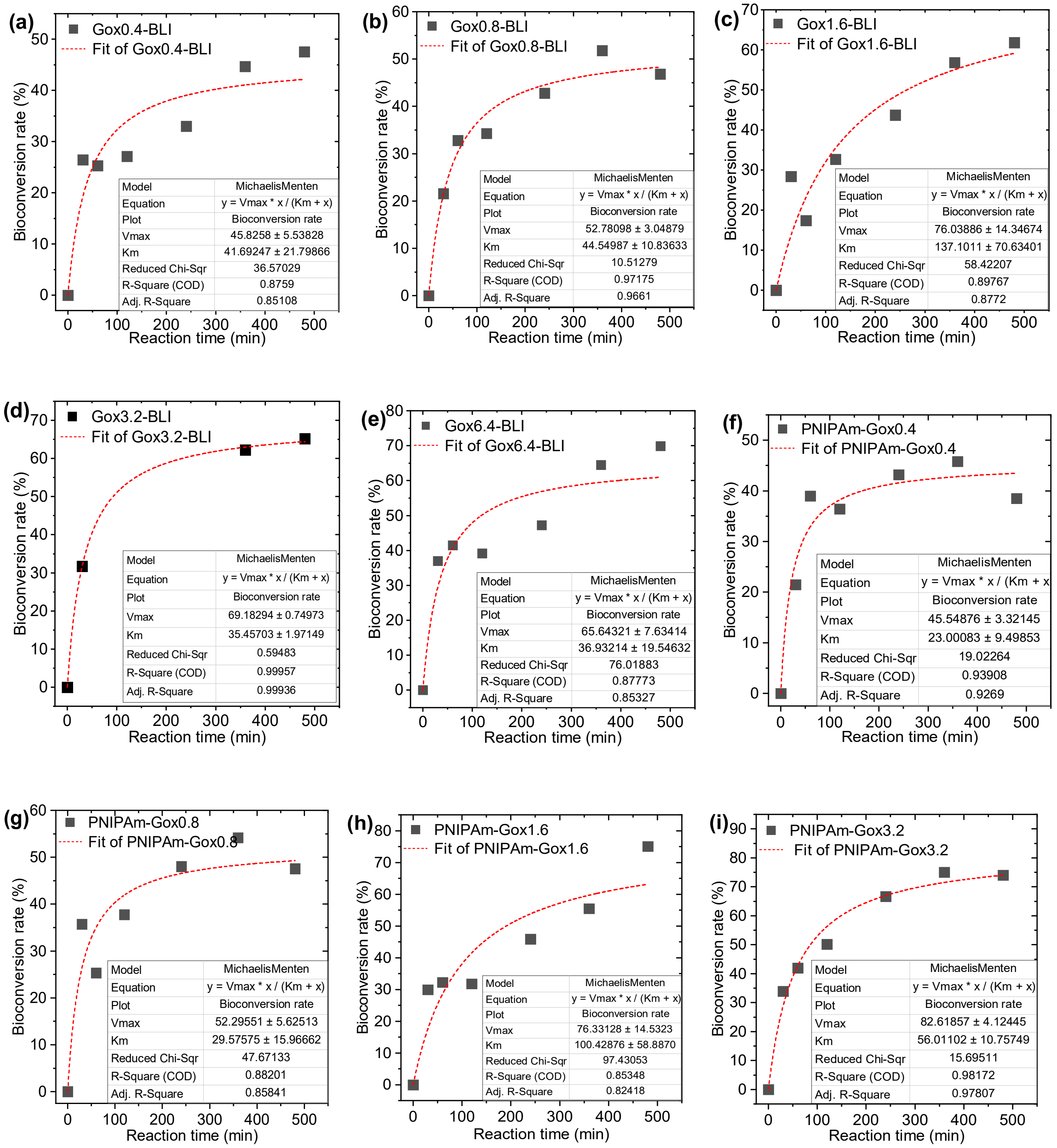
(a)
Gox0.4-BLI
Fit of Gox0.4-BLI
Model MichaelisMenten
Equation y = Vmax * x / (Km + x)
Plot Bioconversion rate
Vmax 45.8258 ± 5.53828
Km 41.69247 ± 21.79866
Reduced Chi-Sqr 36.57029
R-Square (COD) 0.8759
Adj. R-Square 0.85108
Bioconversion rate (%)
Reaction time (min)
(b)
Gox0.8-BLI
Fit of Gox0.8-BLI
Model MichaelisMenten
Equation y = Vmax * x / (Km + x)
Plot Bioconversion rate
Vmax 52.78098 ± 3.04879
Km 44.54987 ± 10.83633
Reduced Chi-Sqr 10.51279
R-Square (COD) 0.97175
Adj. R-Square 0.9661
Bioconversion rate (%)
Reaction time (min)
(c)
Gox1.6-BLI
Fit of Gox1.6-BLI
Model MichaelisMenten
Equation y = Vmax * x / (Km + x)
Plot Bioconversion rate
Vmax 76.03886 ± 14.34674
Km 137.1011 ± 70.63401
Reduced Chi-Sqr 58.42207
R-Square (COD) 0.89767
Adj. R-Square 0.8772
Bioconversion rate (%)
Reaction time (min)
(d)
Gox3.2-BLI
Fit of Gox3.2-BLI
Model MichaelisMenten
Equation y = Vmax * x / (Km + x)
Plot Bioconversion rate
Vmax 69.18294 ± 0.74973
Km 35.45703 ± 1.97149
Reduced Chi-Sqr 0.59483
R-Square (COD) 0.99957
Adj. R-Square 0.99936
Bioconversion rate (%)
Reaction time (min)
(e)
Gox6.4-BLI
Fit of Gox6.4-BLI
Model MichaelisMenten
Equation y = Vmax * x / (Km + x)
Plot Bioconversion rate
Vmax 65.64321 ± 7.63414
Km 36.93214 ± 19.54632
Reduced Chi-Sqr 76.01883
R-Square (COD) 0.87773
Adj. R-Square 0.85327
Bioconversion rate (%)
Reaction time (min)
(f)
PNIPAm-Gox0.4
Fit of PNIPAm-Gox0.4
Model MichaelisMenten
Equation y = Vmax * x / (Km + x
Plot Bioconversion rate
Vmax 45.54876 ± 3.32145
Km 23.00083 ± 9.49853
Reduced Chi-Sqr 19.02264
R-Square (COD) 0.93908
Adj. R-Square 0.9269
Bioconversion rate (%)
Reaction time (min)
(g)
PNIPAm-Gox0.8
Fit of PNIPAm-Gox0.8
Model MichaelisMenten
Equation y = Vmax * x / (Km + x)
Plot Bioconversion rate
Vmax 52.29551 ± 5.62513
Km 29.57575 ± 15.96662
Reduced Chi-Sqr 47.67133
R-Square (COD) 0.88201
Adj. R-Square 0.85841
Bioconversion rate (%)
Reaction time (min)
(h)
PNIPAm-Gox1.6
Fit of PNIPAm-Gox1.6
Model MichaelisMenten
Equation y = Vmax * x / (Km + x)
Plot Bioconversion rate
Vmax 76.33128 ± 14.5323
Km 100.42876 ± 58.8870
Reduced Chi-Sqr 97.43053
R-Square (COD) 0.85348
Adj. R-Square 0.82418
Bioconversion rate (%)
Reaction time (min)
(i)
PNIPAm-Gox3.2
Fit of PNIPAm-Gox3.2
Model MichaelisMenten
Equation y = Vmax * x / (Km + x)
Plot Bioconversion rate
Vmax 82.61857 ± 4.12445
Km 56.01102 ± 10.75749
Reduced Chi-Sqr 15.69511
R-Square (COD) 0.98172
Adj. R-Square 0.97807
Bioconversion rate (%)
Reaction time (min)

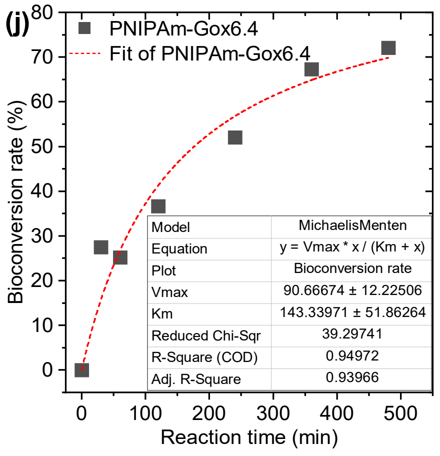

Figure S4. Fit the enzymatic reaction proceed curve of (a) Gox0.4-BLI, (b) Gox0.8-BLI, (c) Gox1.6-BLI, (d) Gox3.2-BLI, (e) Gox6.4-BLI, (f) PNIPAm-Gox0.4, (g) PNIPAm-Gox0.8, (h) PNIPAm-Gox1.6, (i) PNIPAm-Gox3.2, (j) PNIPAm-Gox6.4 by using (Eq.1). Vmax represents the maximum production rate, Km represents the reaction time for produce half of the maximum production rate.

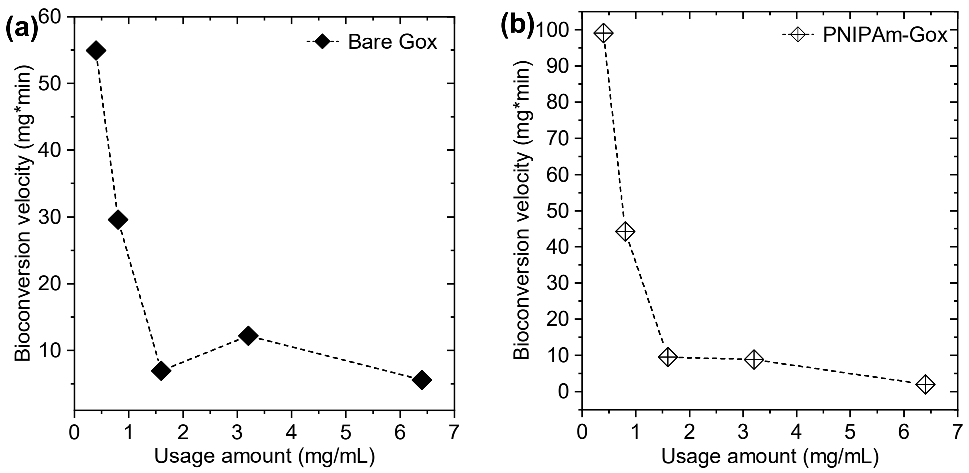

Figure S5. Calculated bioconversion velocity v.s. enzyme protein usage amount of (a) bare Gox after blue light illumination and (b) PNIPAm-Gox.

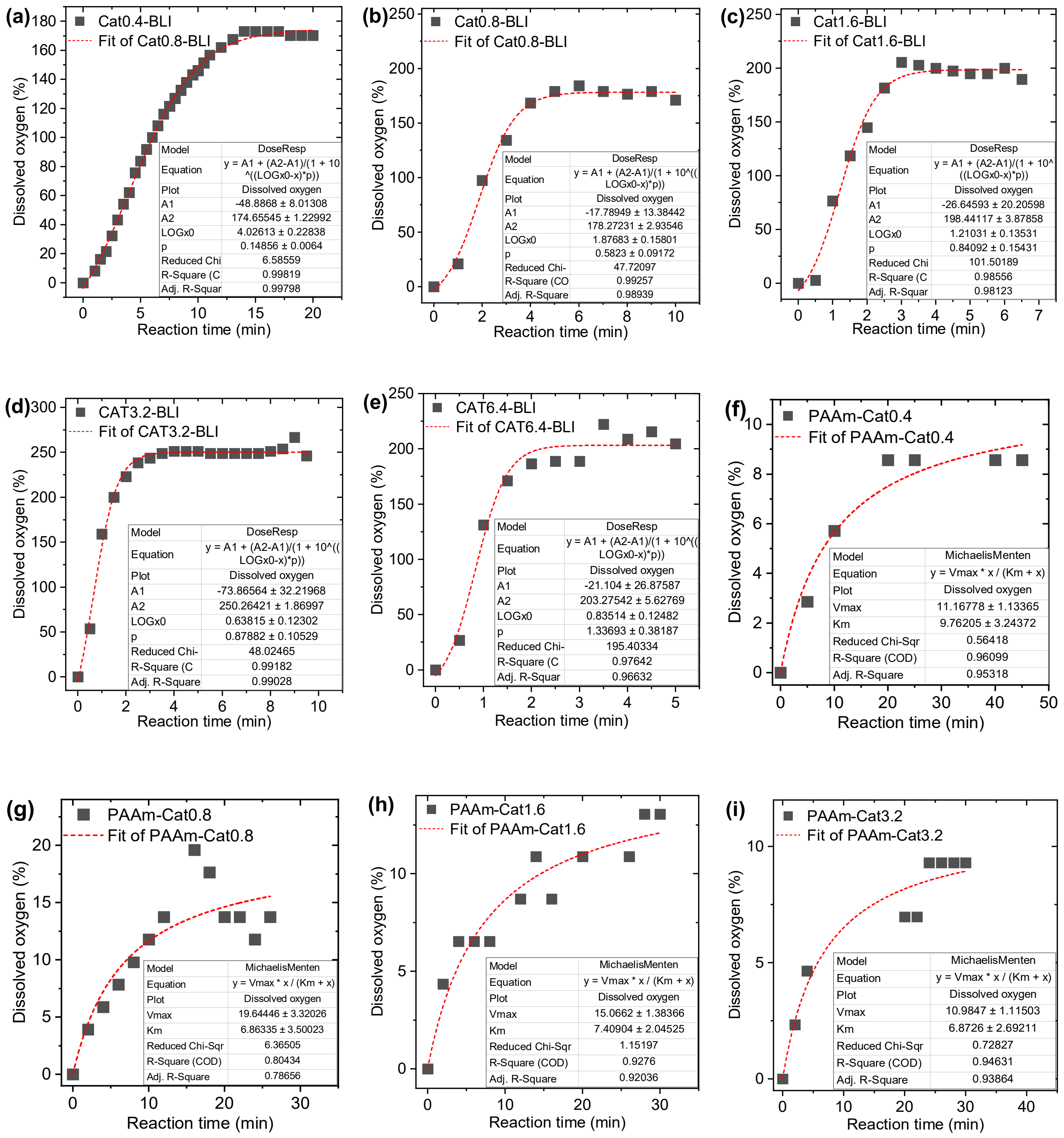
(a)
Cat0.4-BLI
Fit of Cat0.8-BLI
Dissolved oxygen (%)
Reaction time (min)
Model DoseResp
Equation y = A1 + (A2-A1)/(1 + 10^((LOGx0-x)*p))
Plot Dissolved oxygen
A1 -48.8868 ± 8.01308
A2 174.65545 ± 1.22992
LOGx0 4.02613 ± 0.22838
p 0.14856 ± 0.0064
Reduced Chi 6.58559
R-Square (C 0.99819
Adj. R-Squar 0.99798
(b)
Cat0.8-BLI
Fit of Cat0.8-BLI
Dissolved oxygen (%)
Reaction time (min)
Model DoseResp
Equation y = A1 + (A2-A1)/(1 + 10^((LOGx0-x)*p))
Plot Dissolved oxygen
A1 -17.78949 ± 13.38442
A2 178.27231 ± 2.93546
LOGx0 1.87683 ± 0.15801
p 0.5823 ± 0.09172
Reduced Chi- 47.72097
R-Square (CO 0.99257
Adj. R-Square 0.98939
(c)
Cat1.6-BLI
Fit of Cat1.6-BLI
Dissolved oxygen (%)
Reaction time (min)
Model DoseResp
Equation y = A1 + (A2-A1)/(1 + 10^((LOGx0-x)*p))
Plot Dissolved oxygen
A1 -26.64593 ± 20.20598
A2 198.44117 ± 3.87858
LOGx0 1.21031 ± 0.13531
p 0.84092 ± 0.15431
Reduced Chi 101.50189
R-Square (C 0.98556
Adj. R-Squar 0.98123
(d)
CAT3.2-BLI
Fit of CAT3.2-BLI
Dissolved oxygen (%)
Reaction time (min)
Model DoseResp
Equation y = A1 + (A2-A1)/(1 + 10^((LOGx0-x)*p))
Plot Dissolved oxygen
A1 -73.86564 ± 32.21968
A2 250.26421 ± 1.86997
LOGx0 0.63815 ± 0.12302
p 0.87882 ± 0.10529
Reduced Chi- 48.02465
R-Square (C 0.99182
Adj. R-Square 0.99028
(e)
CAT6.4-BLI
Fit of CAT6.4-BLI
Dissolved oxygen (%)
Reaction time (min)
Model DoseResp
Equation y = A1 + (A2-A1)/(1 + 10^((LOGx0-x)*p))
Plot Dissolved oxygen
A1 -21.104 ± 26.87587
A2 203.27542 ± 5.62769
LOGx0 0.83514 ± 0.12482
p 1.33693 ± 0.38187
Reduced Chi- 195.40334
R-Square (C 0.97642
Adj. R-Squar 0.96632
(f)
PAAm-Cat0.4
Fit of PAAm-Cat0.4
Dissolved oxygen (%)
Reaction time (min)
Model MichaelisMenten
Equation y = Vmax * x / (Km + x)
Plot Dissolved oxygen
Vmax 11.16778 ± 1.13365
Km 9.76205 ± 3.24372
Reduced Chi-Sqr 0.56418
R-Square (COD) 0.96099
Adj. R-Square 0.95318
(g)
PAAm-Cat0.8
Fit of PAAm-Cat0.8
Dissolved oxygen (%)
Reaction time (min)
Model MichaelisMenten
Equation y = Vmax * x / (Km + x)
Plot Dissolved oxygen
Vmax 19.64446 ± 3.32026
Km 6.86335 ± 3.50023
Reduced Chi-Sqr 6.36505
R-Square (COD) 0.80434
Adj. R-Square 0.78656
(h)
PAAm-Cat1.6
Fit of PAAm-Cat1.6
Dissolved oxygen (%)
Reaction time (min)
Model MichaelisMenten
Equation y = Vmax * x / (Km + x)
Plot Dissolved oxygen
Vmax 15.0662 ± 1.38366
Km 7.40904 ± 2.04525
Reduced Chi-Sqr 1.15197
R-Square (COD) 0.9276
Adj. R-Square 0.92036
(i)
PAAm-Cat3.2
Fit of PAAm-Cat3.2
Dissolved oxygen (%)
Reaction time (min)
Model MichaelisMenten
Equation y = Vmax * x / (Km + x)
Plot Dissolved oxygen
Vmax 10.9847 ± 1.11503
Km 6.8726 ± 2.69211
Reduced Chi-Sqr 0.72827
R-Square (COD) 0.94631
Adj. R-Square 0.93864

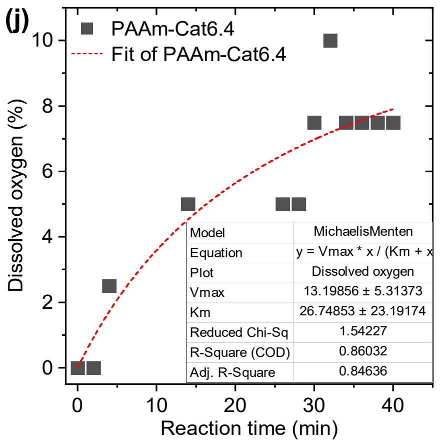

Figure S6. Fit the enzymatic reaction proceed curve of (a) Cat0.4-BLI, (b) Cat0.8-BLI, (c) Cat1.6-BLI, (d) Cat3.2-BLI, (e) Cat6.4-BLI by using (Eq.2), and (f) PAAm-Cat0.4, (g) PAAm-Cat0.8, (h) PAAm-Cat1.6, (i) PAAm-Cat3.2, (j) PAAm-Cat6.4 by using (Eq.1). Vmax represents the maximum production rate, Km represents the reaction time for produce half of the maximum production rate. The 1/2 (A1+A2) was defined as the product volume at maximum production speed, and the $\log x_0$ was defined as the time it takes to produce the product at the maximum speed.

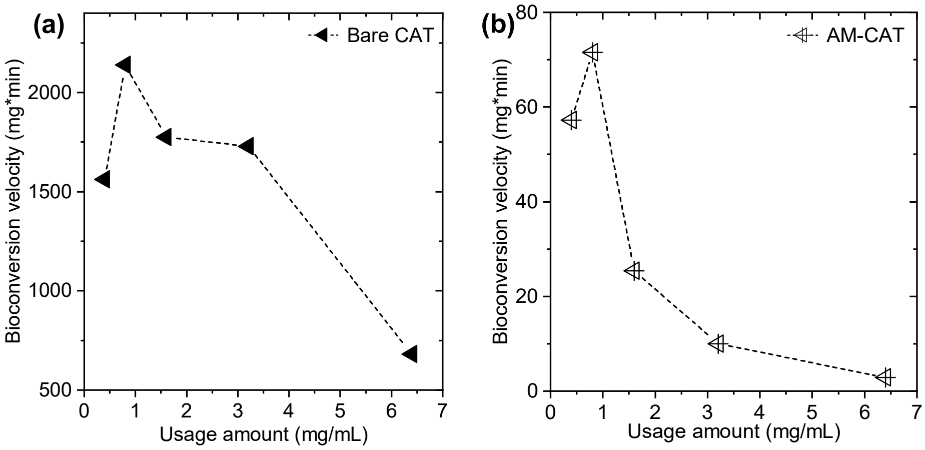

Figure S7. Calculated bioconversion velocity v.s. enzyme protein usage amount of (a) bare Cat after blue light illumination and (b) PAAm-Cat.

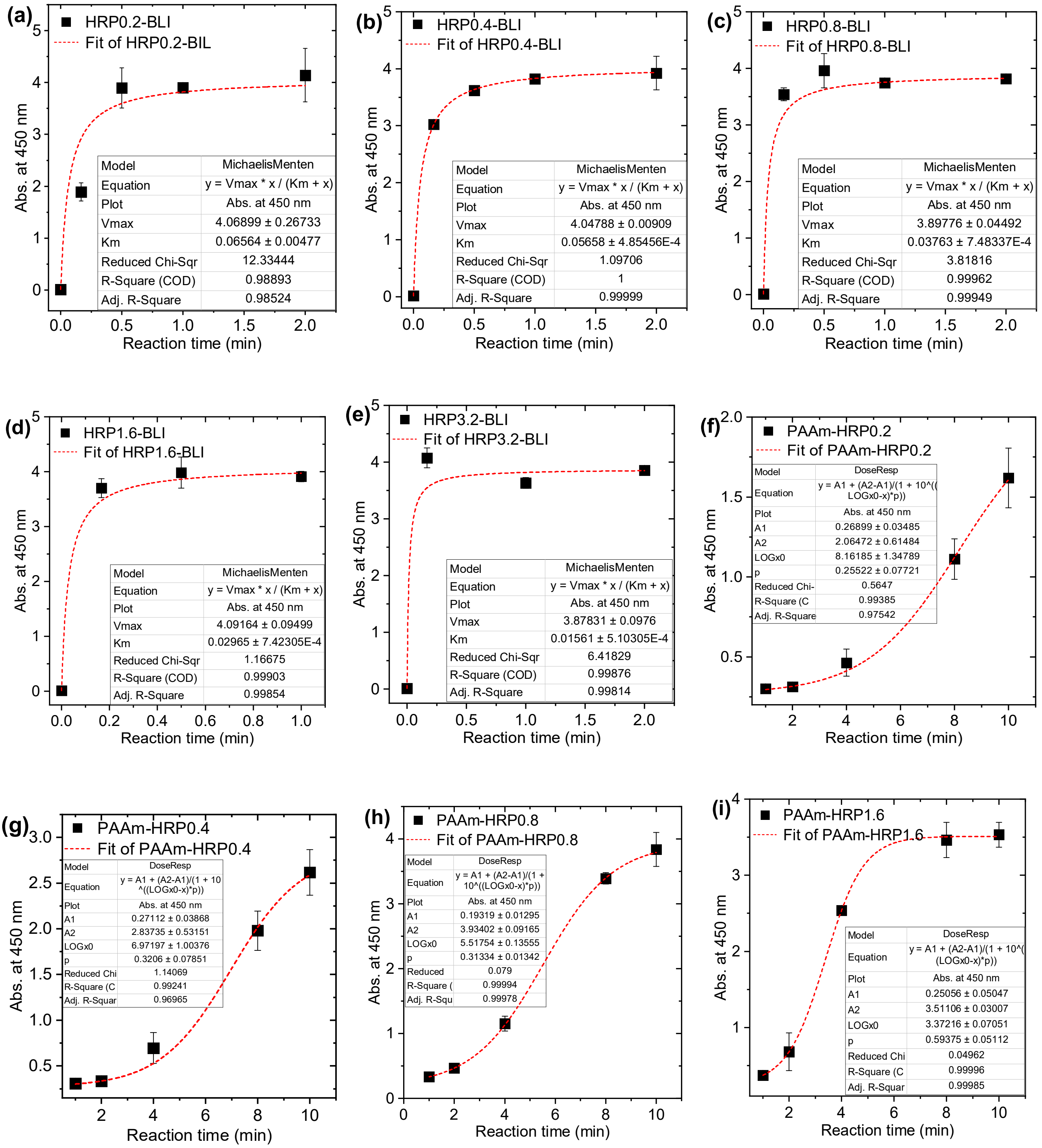
(a)
HRP0.2-BLI
Fit of HRP0.2-BIL
Model MichaelisMenten
Equation y = Vmax * x / (Km + x)
Plot Abs. at 450 nm
Vmax 4.06899 ± 0.26733
Km 0.06564 ± 0.00477
Reduced Chi-Sqr 12.33444
R-Square (COD) 0.98893
Adj. R-Square 0.98524
Abs. at 450 nm
Reaction time (min)
(b)
HRP0.4-BLI
Fit of HRP0.4-BLI
Model MichaelisMenten
Equation y = Vmax * x / (Km + x)
Plot Abs. at 450 nm
Vmax 4.04788 ± 0.00909
Km 0.05658 ± 4.85456E-4
Reduced Chi-Sqr 1.09706
R-Square (COD) 1
Adj. R-Square 0.99999
Abs. at 450 nm
Reaction time (min)
(c)
HRP0.8-BLI
Fit of HRP0.8-BLI
Model MichaelisMenten
Equation y = Vmax * x / (Km + x)
Plot Abs. at 450 nm
Vmax 3.89776 ± 0.04492
Km 0.03763 ± 7.48337E-4
Reduced Chi-Sqr 3.81816
R-Square (COD) 0.99962
Adj. R-Square 0.99949
Abs. at 450 nm
Reaction time (min)
(d)
HRP1.6-BLI
Fit of HRP1.6-BLI
Model MichaelisMenten
Equation y = Vmax * x / (Km + x)
Plot Abs. at 450 nm
Vmax 4.09164 ± 0.09499
Km 0.02965 ± 7.42305E-4
Reduced Chi-Sqr 1.16675
R-Square (COD) 0.99903
Adj. R-Square 0.99854
Abs. at 450 nm
Reaction time (min)
(e)
HRP3.2-BLI
Fit of HRP3.2-BLI
Model MichaelisMenten
Equation y = Vmax * x / (Km + x)
Plot Abs. at 450 nm
Vmax 3.87831 ± 0.0976
Km 0.01561 ± 5.10305E-4
Reduced Chi-Sqr 6.41829
R-Square (COD) 0.99876
Adj. R-Square 0.99814
Abs. at 450 nm
Reaction time (min)
(f)
PAAm-HRP0.2
Fit of PAAm-HRP0.2
Model DoseResp
Equation y = A1 + (A2-A1)/(1 + 10^((LOGx0-x)*p))
Plot Abs. at 450 nm
A1 0.26899 ± 0.03485
A2 2.06472 ± 0.61484
LOGx0 8.16185 ± 1.34789
p 0.25522 ± 0.07721
Reduced Chi- 0.5647
R-Square (C 0.99385
Adj. R-Square 0.97542
Abs. at 450 nm
Reaction time (min)
(g)
PAAm-HRP0.4
Fit of PAAm-HRP0.4
Model DoseResp
Equation y = A1 + (A2-A1)/(1 + 10^((LOGx0-x)*p))
Plot Abs. at 450 nm
A1 0.27112 ± 0.03868
A2 2.83735 ± 0.53151
LOGx0 6.97197 ± 1.00376
p 0.3206 ± 0.07851
Reduced Chi 1.14069
R-Square (C 0.99241
Adj. R-Squar 0.96965
Abs. at 450 nm
Reaction time (min)
(h)
PAAm-HRP0.8
Fit of PAAm-HRP0.8
Model DoseResp
Equation y = A1 + (A2-A1)/(1 + 10^((LOGx0-x)*p))
Plot Abs. at 450 nm
A1 0.19319 ± 0.01295
A2 3.93402 ± 0.09165
LOGx0 5.51754 ± 0.13555
p 0.31334 ± 0.01342
Reduced 0.079
R-Square (0.99994
Adj. R-Squ 0.99978
Abs. at 450 nm
Reaction time (min)
(i)
PAAm-HRP1.6
Fit of PAAm-HRP1.6
Model DoseResp
Equation y = A1 + (A2-A1)/(1 + 10^((LOGx0-x)*p))
Plot Abs. at 450 nm
A1 0.25056 ± 0.05047
A2 3.51106 ± 0.03007
LOGx0 3.37216 ± 0.07051
p 0.59375 ± 0.05112
Reduced Chi 0.04962
R-Square (C 0.99996
Adj. R-Squar 0.99985
Abs. at 450 nm
Reaction time (min)

(j)

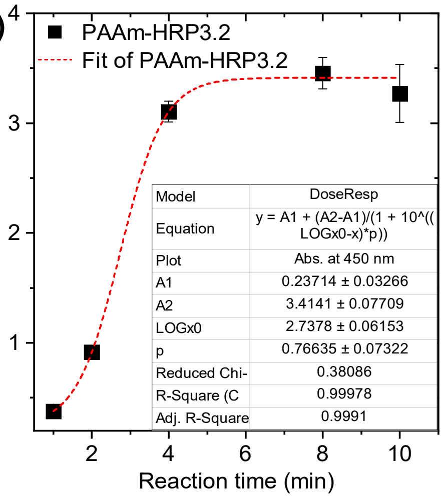

Figure S8. Fit the enzymatic reaction proceed curve of (a) HRP0.4-BLI, (b) HRP0.8-BLI, (c) HRP1.6-BLI, (d) HRP3.2-BLI, (e) HRP6.4-BLI by using (Eq.1), and (f) PAAm-HRP0.4, (g) PAAm-HRP0.8, (h) PAAm-HRP1.6, (i) PAAm-HRP3.2, (j) PAAm-HRP6.4 by using (Eq.2). Vmax represents the maximum production, Km represents the reaction time for produce ½ Vmax. Fit the enzymatic reaction proceed curve of AM-HRP by equation (2). ½ (A1+A2) represents the product volume at maximum production speed, LOGx0 represents the time it takes to produce the product at the maximum speed.

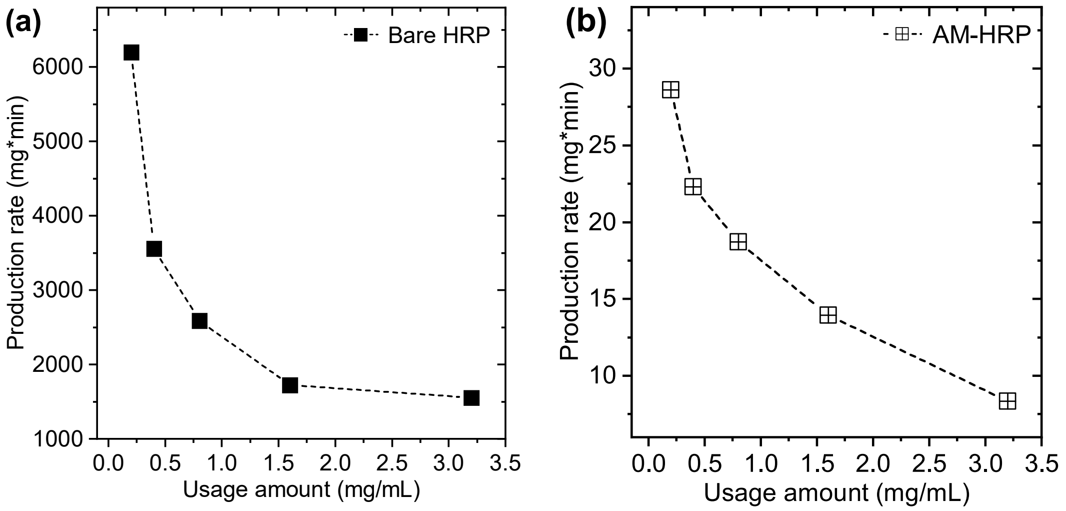

Figure S9. Bioconversion rate of HRP-BIL and Am-HRP with different usage amount of (a) bare HRP after blue light illumination and (b) PAAm-HRP.
